# Phylogeny and evolutionary history of glycogen synthase kinase 3/SHAGGY-like kinase genes in land plants

**DOI:** 10.1186/1471-2148-13-143

**Published:** 2013-07-08

**Authors:** Xinshuai Qi, André S Chanderbali, Gane Ka-Shu Wong, Douglas E Soltis, Pamela S Soltis

**Affiliations:** 1 Key Laboratory of Conservation Biology for Endangered Wildlife of the Ministry of Education, College of Life Sciences, Zhejiang University, Hangzhou, China; 2 Laboratory of Systematic & Evolutionary Botany and Biodiversity, College of Life Sciences, Zhejiang University, Hangzhou, China; 3 Department of Biology, University of Florida, Gainesville, FL, USA; 4 Florida Museum of Natural History, University of Florida, Gainesville, FL, USA; 5 Department of Biological Sciences, University of Alberta, Edmonton, AB, Canada; 6 Department of Medicine, University of Alberta, Edmonton, AB, Canada; 7 BGI-Shenzhen, Bei Shan Industrial Zone, Yantian District, Shenzhen, China

**Keywords:** *GSK3*, Land plant evolution, Gene duplication, Gene expression

## Abstract

**Background:**

*GSK3* (*glycogen synthase kinase 3*) genes encode signal transduction proteins with roles in a variety of biological processes in eukaryotes. In contrast to the low copy numbers observed in animals, *GSK3* genes have expanded into a multi-gene family in land plants (embryophytes), and have also evolved functions in diverse plant specific processes, including floral development in angiosperms. However, despite previous efforts, the phylogeny of land plant *GSK3* genes is currently unclear. Here, we analyze genes from a representative sample of phylogenetically pivotal taxa, including basal angiosperms, gymnosperms, and monilophytes, to reconstruct the evolutionary history and functional diversification of the *GSK3* gene family in land plants.

**Results:**

Maximum Likelihood phylogenetic analyses resolve a gene tree with four major gene duplication events that coincide with the emergence of novel land plant clades. The single *GSK3* gene inherited from the ancestor of land plants was first duplicated along the ancestral branch to extant vascular plants, and three subsequent duplications produced three *GSK3* loci in the ancestor of euphyllophytes, four in the ancestor of seed plants, and at least five in the ancestor of angiosperms. A single gene in the *Amborella trichopoda* genome may be the sole survivor of a sixth *GSK3* locus that originated in the ancestor of extant angiosperms. Homologs of two *Arabidopsis GSK3* genes with genetically confirmed roles in floral development*, AtSK11* and *AtSK12,* exhibit floral preferential expression in several basal angiosperms, suggesting evolutionary conservation of their floral functions. Members of other gene lineages appear to have independently evolved roles in plant reproductive tissues in individual taxa.

**Conclusions:**

Our phylogenetic analyses provide the most detailed reconstruction of *GSK3* gene evolution in land plants to date and offer new insights into the origins, relationships, and functions of family members. Notably, the diversity of this “green” branch of the gene family has increased in concert with the increasing morphological and physiological complexity of land plant life forms. Expression data for seed plants indicate that the functions of *GSK3* genes have also diversified during evolutionary time.

## Background

Glycogen synthase kinase 3 (GSK3) proteins, also known as SHAGGY-like kinases, have important roles in a wide range of cellular processes throughout eukaryotes [1]. In animal development, products of *GSK3* homologs participate in the critically important Wnt signaling pathway that regulates cellular differentiation, patterning, and growth in perhaps all metazoans [2]. The *GSK3* homolog in the protozoan *Dictyostelium discoideum* is also involved in the regulation of development [3]. Recognition of possible roles of *GSK3* in human disease has prompted recent interest in these genes in the field of medicine [1,4]. Compared to animals, *GSK3* genes have radiated into a relatively large multi-gene family in land plants [5-7]. For example, five *GSK3* genes have been reported from the moss *Physcomitrella patens*[8], and 10 *GSK3* genes are present in the genome sequence of the flowering plant *Arabidopsis thaliana*[5]. Conceivably, therefore, *GSK3* genes have had a dynamic history of gene duplication during the course of land plant evolution. They have also acquired roles in plant-specific processes. For example, different *Arabidopsis GSK3* genes function in hormonal signaling, osmotic stress responses [1], and flower development [6,9].

Previous phylogenetic analyses suggest that four major lineages evolved in the land plant branch of the *GSK3* gene family [7], but their origins and relationships are currently unclear. *Physcomitrella GSK3* genes occupy different positions relative to groups of angiosperm genes in various analyses [8,10], and the positions of fern and gymnosperm *GSK3* sequences have been similarly fluid (Figures five to seven in [10]). Such topological instabilities may be an indication of inadequate sampling, which is often a problem in phylogenetic reconstruction [11], particularly in gene family analyses in which both taxonomic and gene copy representation may be sparse. For example, only three plant genomes were available to Yoo et al. [10], and ferns and gymnosperms were represented by just seven sequences in their data set. Currently, 35 land plant genomes are publically accessible through the Phytozome v9.0 portal [12]. We also have a draft genome sequence for *Amborella trichopoda*, which occupies a pivotal phylogenetic position as sister to all other extant flowering plants [13]. In addition, the Ancestral Angiosperm Genome (http://ancangio.uga.edu/) and 1KP (http://www.onekp.com/project.html) projects provide transcriptome assemblies for taxa representing lineages that are critical for understanding gene family evolution in land plants; mosses, liverworts, lycophytes, monilophytes, and gymnosperms, as well as angiosperms.

Here, we use the newly available genomic resources reviewed above to reconstruct the phylogenetic history of *GSK3* genes in land plants (embryophytes). We include sequences of two chlorophyte algae as outgroups. Specifically, we: (1) clarify the phylogenetic relationships among land plant *GSK3* genes via our greatly increased taxon sampling, (2) reconstruct the history of gene duplication and extinction during land plant diversification, and (3) identify shifts in tissue-preferential expression that may relate to functional diversification in seed plants.

## Results and discussion

**Figure 1:**
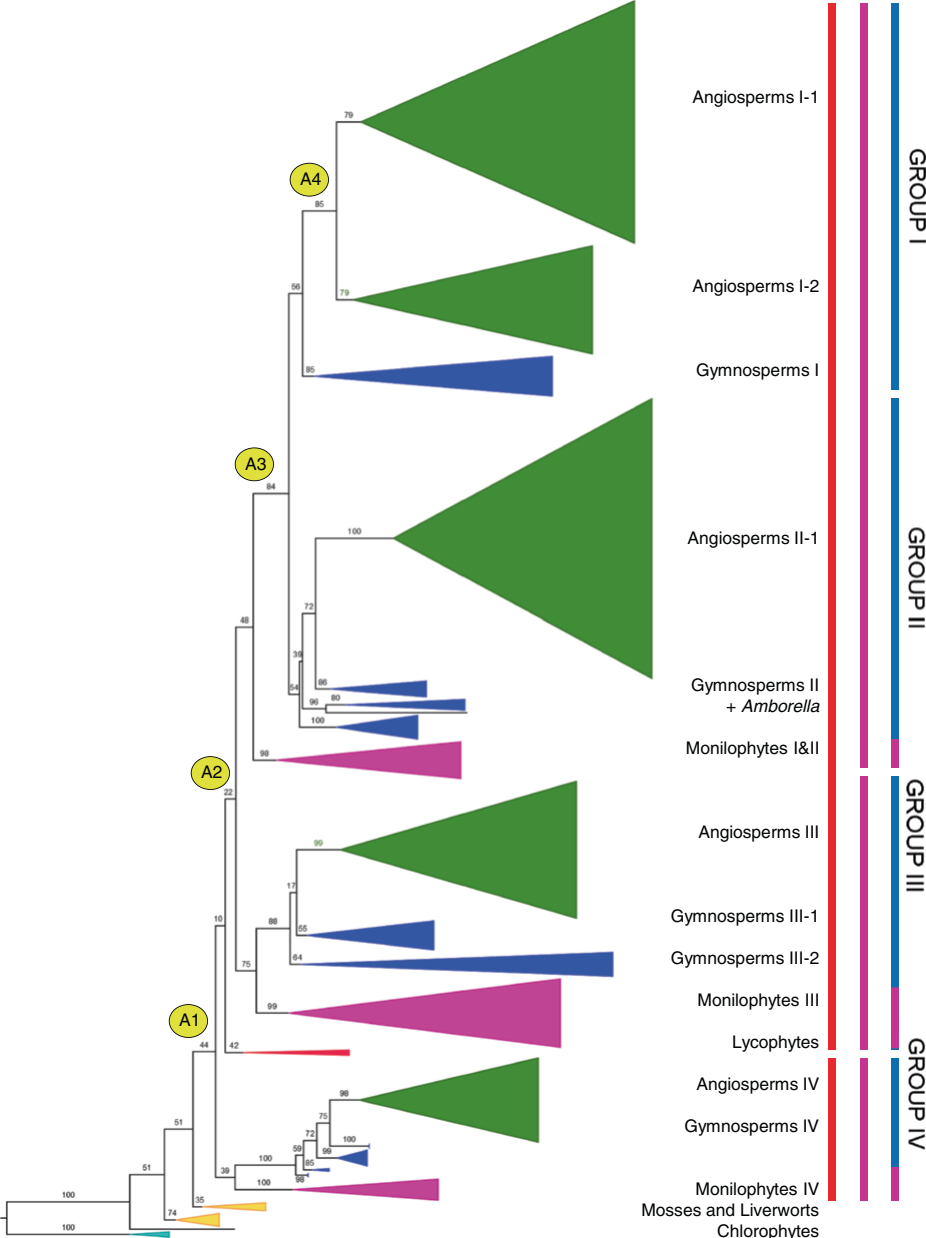
**Phylogenetic relationships among land plant *GSK3 *genes.** Four major groups (I - IV) and four major gene duplication events (A1 - A4) are recognized. Numbers above branches indicate bootstrap support values (%). Gene lineages composed of angiosperms, gymnosperms, monilophytes, lycophytes, liverworts, mosses, and algae are labeled. Color bars to the right demarcate gene lineages that originated in the ancestors of extant tracheophytes (red), euphyllophytes (purple), and seed plants (green).

**Figure 2:**
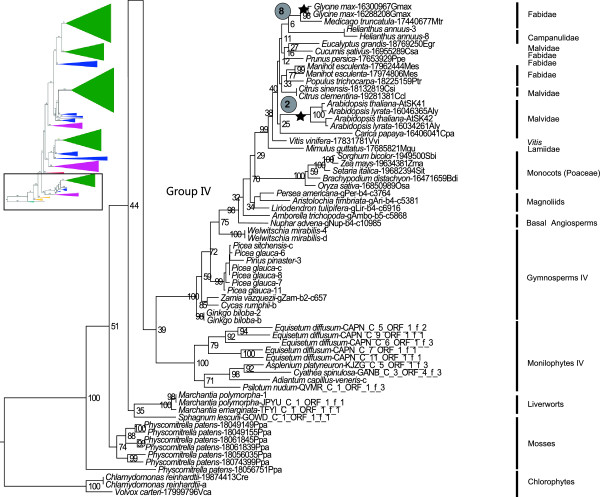
**Details of phylogenetic relationships among basal land plant *GSK3 *genes and within the Group IV gene lineage.** Upper left insert indicates the position of the depicted phylogeny relative to the overall land plant *GSK3* gene tree depicted in Figure 1. Stars correspond to postulated whole-genome duplication events in Table 1. Bootstrap support values are provided adjacent to nodes.

**Figure 3:**
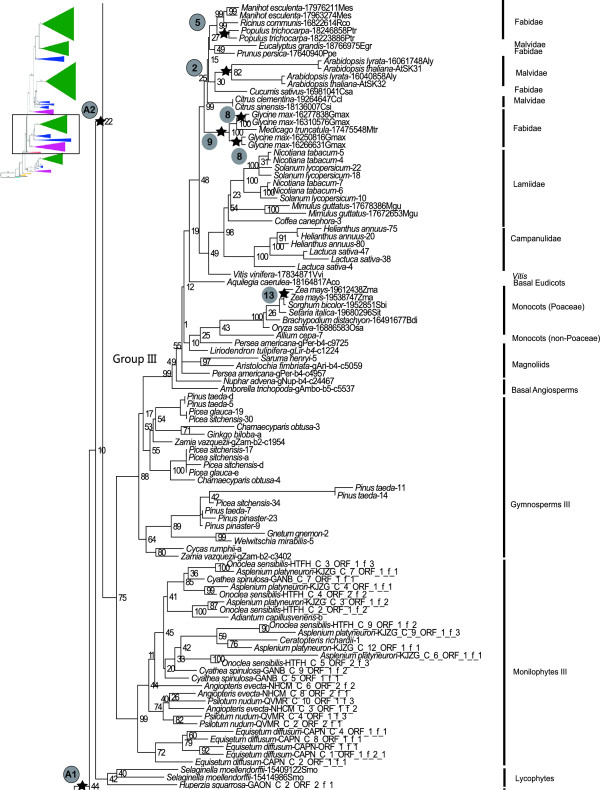
**Phylogenetic position of lycophytes, and relationships among Group III *GSK3 *genes.** Upper left insert indicates the position of the depicted phylogeny relative to the overall land plant *GSK3* gene tree depicted in Figure 1. Stars correspond to postulated whole-genome duplication events in Table 1. Bootstrap support values are provided adjacent to nodes.

**Figure 4:**
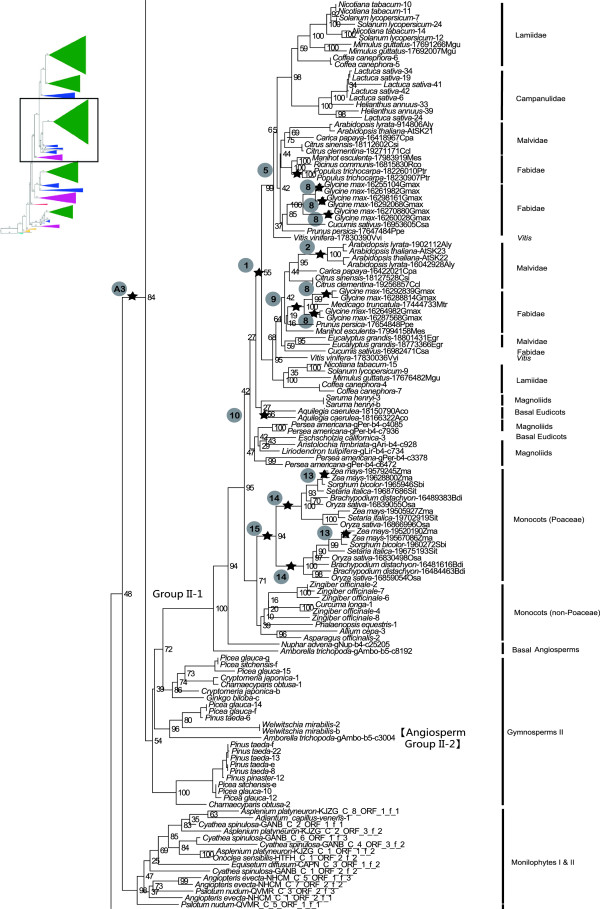
**Phylogenetic relationships within Monilophyte I** &**II and among Group II *GSK3 *genes.** Upper left insert indicates the position of the depicted phylogeny relative to the overall land plant *GSK3* gene tree depicted in Figure 1. Stars correspond to postulated whole-genome duplication events in Table 1. Bootstrap support values are provided adjacent to nodes.

**Figure 5:**
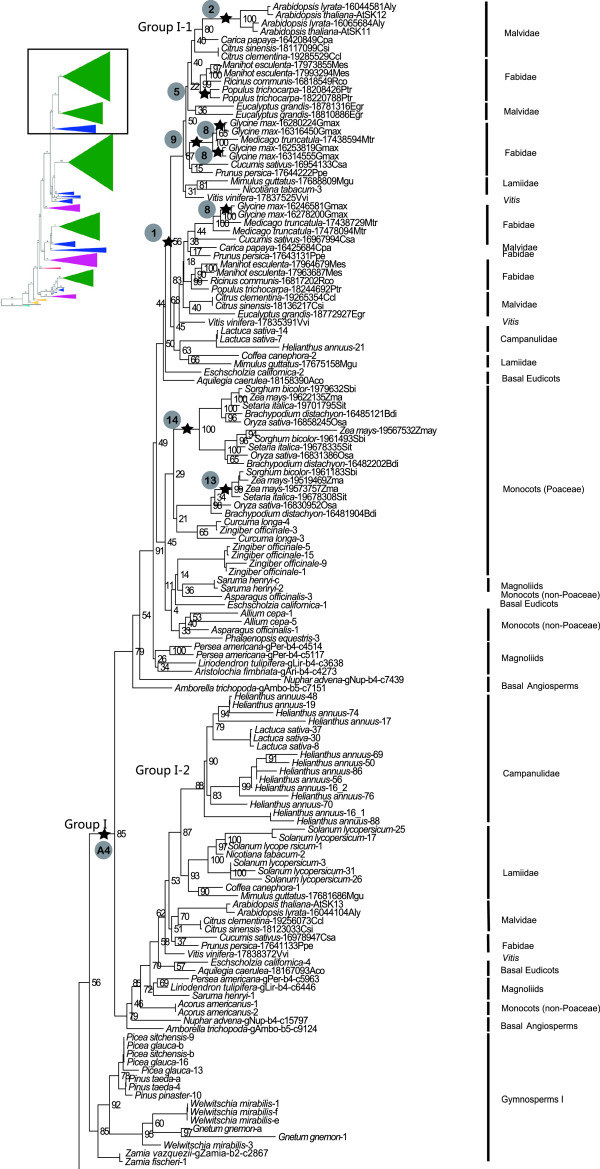
**Phylogenetic relationships among Group I *GSK3 *genes.** Upper left insert indicates the position of the depicted phylogeny relative to the overall land plant *GSK3* gene tree depicted in Figure 1. Stars correspond to postulated whole-genome duplication events numbered in Table 1. Bootstrap support values are provided adjacent to nodes.

Our Maximum Likelihood phylogeny of land plant *GSK3* genes (schematic summary in Figure 1, details in Figures 2, 3, 4, 5), rooted with the chlorophyte algae *Volvox* and *Chlamydomonas*, is largely congruent with established organismal relationships (e.g. [14,15]). The basal branches constitute a grade of “bryophyte” sequences, above which the tree topology reveals three ancient gene duplication events along the branches leading to extant tracheophytes (vascular plants), euphyllophytes (monilophytes and seed plants), and spermatophytes (seed plants), respectively (A1-A3, Figure 1). These duplication events together produced four groups of seed plant genes that correspond with the gene groups previously identified in *Arabidopsis*[5,10]. A subsequent duplication along the branch leading directly to extant angiosperms (A4, Figure 1) produced additional angiosperm-wide gene lineages that we designate as subgroups.

### Ancestral *GSK3* copy number in land plants

At the base of the tree, a single gene from the moss *Physcomitrella* is sister to all other land plant genes, and successive branches lead to a clade of six other genes in the *Physcomitrella* genome, followed by a clade in which a single *Sphagnum* (moss) sequence is sister to three sequences from two *Marchantia* species (liverworts). The branching sequence among these genes implies a duplication event in the ancestral lineage of land plants, with one of the two descendant lineages surviving as a single gene only in *Physcomitrella*. However, since the available sampling of mosses and liverworts is relatively sparse, the present topology might not accurately represent gene phylogeny. Therefore, origin of the isolated *Physcomitrella* gene through a more recent duplication, perhaps on the branch leading directly to *Physcomitrella* or extant mosses, remains feasible.

### Duplication along the ancestral branch to tracheophytes

The first duplication in the land plant lineage of *GSK3* genes appears to have occurred along the ancestral branch to tracheophytes (A1 in Figure 1), a clade that emerged during the Silurian period about 415 mya [16]. This “tracheophyte duplication” produced sister gene lineages (orange bars in Figure 1) whose subsequent histories have resulted in disproportionate representation among extant taxa. The larger descendant lineage includes three of the four groups of seed plant *GSK3* genes (I, II, and III), and sequences from *Selaginella* and *Huperzia* (lycophytes) are sister to all euphyllophyte genes. Its sister lineage, which includes the Group IV *GSK3* genes, must have also originated along the ancestral branch to tracheophytes, but does not include lycophyte sequences (Figures 1 and 2). An alternative scenario in which lycophyte genes are placed sister to all euphyllophyte genes, shifting the A1 duplication to the ancestral branch to euphyllophytes, was rejected by an Approximately Unbiased (AU) test [17], P = 0.0003. Therefore, the Group IV *GSK3* gene lineage has been lost from lycophytes sometime during their evolutionary history. The *Selaginella* genome lacks a Group IV gene, but since the transcriptome data for *Huperzia* may not be exhaustive, it is still unclear whether the gene loss event pre-dates lycophyte diversification.

### Duplication along the ancestral branch to euphyllophytes

The two loci produced by the “tracheophyte duplication” have evolved into three euphyllophyte-wide gene lineages (purple bars in Figure 1). Two of these, Group I+II and III) share an immediate sister relationship and therefore originated through a duplication event along the ancestral branch of the euphyllophytes (A2 in Figure 1). The single euphyllophyte-wide gene lineage in the collective sister group of Groups I+II and III, Group IV (Figures 1 and 3), suggests that the above duplication affected only one of the duplicate loci in the euphyllophyte ancestor. A more global duplication event, for example an euphyllophyte whole-genome duplication (WGD), is a less parsimonious scenario that requires loss of one Group IV lineage, prior to the diversification of extant euphyllophytes.

### Duplication(s) along ancestral branch to spermatophytes

Of the three *GSK3* loci present in the euphyllophyte ancestor, at least one was subsequently duplicated on the ancestral branch of seed plants, producing four *GSK3* gene lineages (demarcated by green bars in Figure 1). This duplication event (A3) is unambiguously inferred by the immediate sister relationship between two lineages of seed plant genes, Groups I and II, which are collectively sister to a clade of monilophyte genes (Figures 1 and 4). The A3 duplication coincides with the proposed WGD in the ancestral lineage of extant seed plants, ~320 Ma ago [18], and a synchronous duplication event may have affected the ancestral Group III locus in the seed plant ancestor. The phylogeny of seed plant Group III genes resolves as a single angiosperm lineage (Angiosperm III) and two gymnosperm lineages (Gymnosperm III-1 and III-2) that are paraphyletic with respect to Angiosperm III (Figures 1 and 3). Gymnosperm III-1 includes representatives of all extant gymnosperm lineages (cycadophytes, *Ginkgo*, gnetophytes and pinophytes), while Gymnosperm III-2 lacks gnetophytes (possibly a sampling artifact), and is sister to the Angiosperm III gene clade (Figure 4). The gene tree therefore implies a gene duplication event on the branch to extant seed plants with subsequent loss of one descendant lineage along the branch leading to extant angiosperms. This inferred Group III “seed plant duplication” genes would be congruent with that in its sister clade (which produced Groups I and II), increasing the likelihood of a WGD influencing both events. However, the third gene lineage inherited by seed plants from the euphyllophyte ancestor, Group IV, contains no clear evidence of a seed plant WGD. Here, the gene tree resolves as five sequential branches leading to representatives of the ginkgophytes, cycadophytes, pinophytes, gnetophytes, and angiosperms, respectively (Figure 2).

### Duplication on the ancestral branch to angiosperms

Sister angiosperm-wide gene lineages (Angiosperm I-1 and I-2) imply duplication of the ancestral Group I locus along the branch to extant flowering plants with retention of both duplicate copies (A4 in Figures 1 and 5). This duplication coincides with a proposed WGD event that occurred between 300–192 Mya along the ancestral lineage of angiosperms [18]. Synchronous “angiosperm” duplications are not obvious for the other angiosperm *GSK3* gene lineages, but the two *Amborella* genes in the Group II clade may be noteworthy. One occupies the expected position at the base of a pan-angiosperm gene lineage (Angiosperm II-1), while the other is placed within a paraphyletic group of gymnosperm genes (Gymnosperm II) (Figure 4). The paraphyly of Gymnosperm II is primarily due to three clades of sequences from conifers, two of which are sister to *Ginkgo* and gnetophyte sequences, respectively. This topology may be an artifact of inadequate sampling of non-conifer genes rather than a representation of the true gene tree. The placement of an *Amborella* gene among Gymnosperm II sequences may also be an artifact of phylogeny reconstruction. Otherwise, the gene tree implies that three seed plant clades exist among these Group II genes; one with broad angiosperm and gymnosperm representation, a second represented in *Amborella* and gymnosperms, and the third represented only in conifers. Perhaps instead, two angiosperm lineages (II-1 and II-2) originated through a single gene duplication event along the branch to extant angiosperms, followed by loss of the Angiosperm II-2 lineage after the separation of *Amborella* from other flowering plants. On the basis of the other 22 completely sequenced nuclear genomes in our sample (Additional file 1), the Angiosperm II-2 gene lineage would have become extinct early in angiosperm evolution, certainly prior to the divergence of monocots and eudicots. The placement of the surviving *Amborella* Angiosperm II-2 gene among the Gymnosperm II genes, instead of their sister, could therefore be interpreted as an artifact of phylogeny reconstruction, rather than a reflection of true relationship.

### Gene family expansion in individual land plant lineages

We have identified seven *GSK3* genes in the genome sequence of *Physcomitrella*, two more than previously reported [10], indicating a dramatic increase in gene family members over the course of moss evolution. The genome of the lycophyte *Selaginella* contains only two *GSK3* loci, but a gene loss event may have contributed to this condition (see above). Gene duplication and extinction events are also evident during the diversification of individual euphyllophyte lineages.

#### Monilophytes

Clades of five *Equisetum diffusum* sequences are present in both Groups IV and III genes (Figures 2 and 3), indicating multiple duplications affecting *GSK3* loci in this species. Similarly, multiple clades of *Asplenium platyneuron*, *Cyathea spinulosa*, and *Onoclea sensibilis* sequences in Monilophytes I +II and III (Figures 3 and 4) indicate duplications in these leptosporangiate ferns. These duplication events in *GSK3* gene lineages are consistent with the widely recognized role of polyploidy in the evolutionary history of monilophyte taxa [19].

#### Gymnosperms

The gymnosperms *Ginkgo biloba*, *Picea glauca*, and *Welwitschia mirabilis* each possess duplicate Group IV *GSK3* genes (Figure 2) likely derived from separate duplication events unique to their respective lineages. Relatively recent duplications are evident for the Pinaceae in both clades of Gymnosperm III genes (Figure 3). The Gymnosperm II lineage includes three clades of sequences representing conifers (Figure 4), but uncertainty regarding the relationships of these genes relative to other gymnosperm taxa obscures their evolutionary origin. All Group I gymnosperm sequences form a clade (Gymnosperm I), with separate duplications in gnetophytes, Pinaceae, and *Zamia vazquezii* (Figure 5).

#### Angiosperms

Among the Angiosperm IV group (Figure 2), duplications in *Arabidopsis* and *Glycine max* coincide with postulated WGD events for these taxa [20,21] (Table 1). Duplications are also evident in *Helianthus annuus*, perhaps reflecting an ancient WGD in Heliantheae [22], and *Manihot esculenta*, which has not been associated with polyploidy. Group IV genes have not been found among non-Poaceae monocots, possible reflecting a sampling artifact, but their absence in the sequenced genome of a member of the Ranunculaceae (*Aquilegia caerulea*) and in extensive transcriptome data for *Eschscholzia californica* (Papaveraceae) indicates a gene loss event early during the diversification of the Ranunculales.

**Table 1:** **Duplication events muliplying *GSK3 *gene lineages during seed plant diversification coincide with postulated whole-genome duplication events**

**Ancient duplication**	**Name of the WGD**	**GSK3 clades with evidence of WGD**			
		**IV**	**III**	**I**	**II**
1★	Eudicot hexaploidy	**-**	**-**	**√**	**√**
2★	*Arabidopsis* alpha	**√**	**√**	**√**	**√**
3★	*Arabidopsis* beta	**-**	**-**	**-**	**-**
4★	*Brassica* hexaploidy	**-**	**-**	**-**	**-**
5★	Poplar tetraploidy	**-**	**√**	**√**	**√**
7★	Apple tetraploidy	**-**	**-**	**-**	**-**
8★	Soybean tetraploidy	**√**	**√**	**√**	**√**
9★	Legume tetraploidy	**-**	**√**	**√**	**√**
10★	Columbine tetraploidy	**-**	**-**	**-**	**√**
11★	Flowering plant tetraploidy	**-**	**-**	**√**	**√**
12★	Seed Plant tetraploidy	**-**	**√**	**-**	**-**
13★	Grass tetraploidy	**-**	**√**	**√**	**√**
14★	Monocot tetraploidy A	**-**	**-**	**√**	**√**
15★	Monocot tetraploidy B	**-**	**-**	**-**	**√**

Similar gene loss events are not apparent in the Angiosperm III gene lineage, and, instead, duplications are prominent (Figure 3). These have occurred in both asterid and rosid taxa (i.e., *Arabidopsis*, *Helianthus annuus*, *Lactuca sativa*, legumes, *Manihot esculenta*, *Populus trichocarpa,* and Solanaceae), as well as the monocot *Zea mays*, and several coincide with postulated WGD events (Table 1). As discussed above, one descendant lineage of the Group II “angiosperm” duplication has been almost completely lost but its sister lineage has diversified extensively in angiosperms. In this gene-rich lineage, 38 species encode at least 107 *GSK3* genes with duplications coinciding with nine of the 15 postulated WGD events, including the core eudicot “hexaploidy” event [likely two closely placed WGD; 19, 21, 22] (Figure 4). The two subclades of Angiosperm I genes have had contrasting evolutionary histories (Figure 5). All major angiosperm lineages are represented in the Angiosperm I-1 lineage, but the I-2 lineage was apparently lost early in the evolution of the monocots. Only *Acorus*, the sister group of other monocots, is represented in this lineage. The precise timing of this gene loss remains to be determined, but absence from available sequenced monocot genomes (i.e., *Brachypodium distachyon*, *Oryza sativa*, *Setaria italica*, *Sorghum bicolor*, and *Zea mays*) indicates loss prior to the origin of the Poaceae. More globally, the Angiosperm I-1 lineage has experienced a dramatic expansion in gene copy number relative to Angiosperm I-2, and seven duplication events coincide with postulated WGD events during angiosperm diversification (Table 1). For example, the WGD responsible for the ancestral “hexaploidy” of core eudicots [23-25] is evident as duplicate clades including *Vitis vinifera*, *Fabidae*, *Malvidae*, *Lamiideae*, and *Campanulidae*. As no such duplications are evident in Angiosperm I-2, widespread loss of duplicates must have followed WGD in this gene lineage.

### Evolution of GSK3 gene expression in seed plants

**Figure 6:**
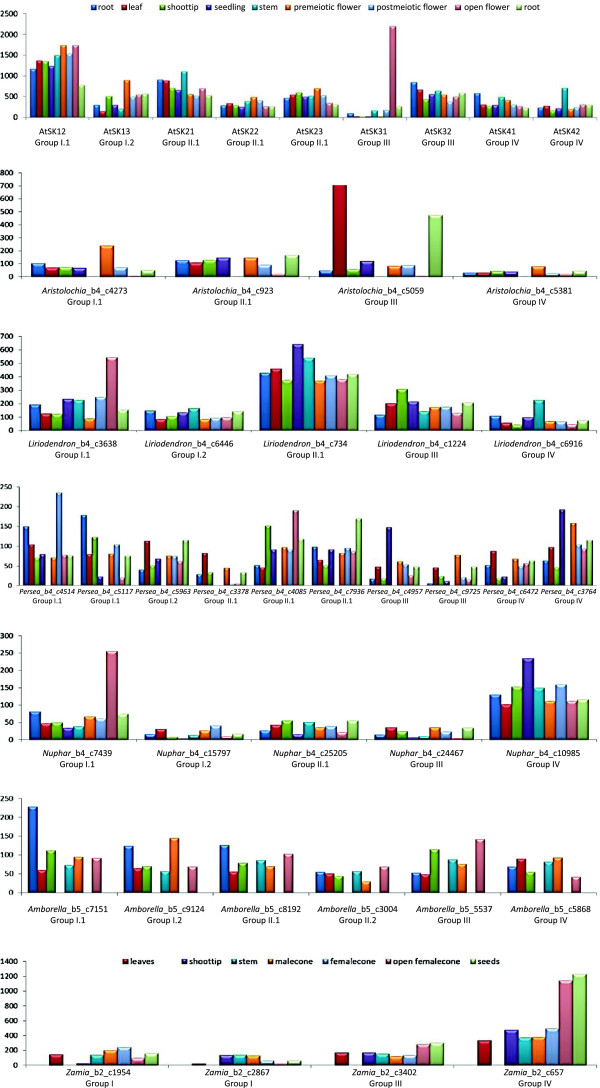
**Expression profiles of *GSK3 *genes across floral and vegetative plant tissues in representative seed plants.** Histograms compare gene expression levels in root, stem, seedling, leaf, flower/cone (during pre-meiotic, post-meiotic, and post-anthetic developmental stages), and fruit/seed of each species, where available. Column heights indicate normalized RPKM values per tissue, except for *Arabidopsis* genes where they indicate normalized microarray signal intensities per tissue.

To assess the evolution of *GSK3* gene expression and, by inference, function, we examined gene expression levels in a functionally diverse set of tissues, including roots, aerial vegetative shoots, and reproductive organs, in six seed plant species (Figure 6). Members of most *GSK3* gene lineages are expressed in almost all tissues examined, supporting their involvement in a wide variety of biological processes. However, these expression data also reveal several instances of tissue preferential expression that suggest roles in specific developmental programs.

Of the three *Arabidopsis* genes implicated in floral development, the roles of Group I genes, *AtSK11* and *AtSK12*, have been confirmed genetically [6], Notably, Group I-1genes from *Aristolochia*, *Liriodendron*, *Persea*, and *Nuphar* exhibit floral-preferential expression, suggesting that the floral function of *AtSK11* and *AtSK12* (recent duplicates in Group I-1) may be evolutionarily conserved in most angiosperms. The *Amborella* Group I-1 gene is also expressed in flowers, but appears to be preferentially expressed in roots. Similarly, Group I genes from *Zamia* are expressed at comparable levels in both vegetative and reproductive tissues. These expression patterns suggest an evolutionary conserved shift to flower preferential expression by Group I-1 GSK3 genes after the divergence of *Amborella* from other angiosperms.

Seedling lethality in mutants [26] have obscured the precise function of *AtSK31* (a Group III gene). According to the microarray data, *AtSK31* is specifically expressed during the latter stages of floral development, where it is closely associated with pollen development in mature stamens [27]. Other Group III genes do not exhibit congruent expression patterns. The *Aristolochia* homolog of *AtSK31* is up-regulated in leaves as well as fruits, but all other angiosperm Group III genes, including *AtSK32* (the paralog of *AtSK31*), show relatively even expression levels across multiple floral and vegetative tissues. The floral function of *AtSK31* is therefore likely to be an example of neo-functionalization after the duplication event that produced paralogous Group III loci in *Arabidopsis*. Approximately two-fold up-regulation of a *Zamia* Group IV gene in open female cones and seeds relative to other tissues may represent another example of independent recruitment of a *GSK3* gene to a role in reproduction; in a gymnosperm in this instance.

## Conclusions

The diversification of the land plant branch of the *GSK3* gene family has been reconstructed in unprecedented detail by our phylogenetic analyses. Four ancient gene duplication events are inferred: in chronological sequence, they occurred along the ancestral branches leading to extant tracheophytes, euphyllophytes, seed plants, and flowering plants, respectively. If these gene duplications were always the result of WGD events, the expected increase in gene lineages was typically countered by loss of at least one descendant lineage. Local duplications affecting single ancestral loci could also explain the asymmetric gene tree topology that our phylogenetic analyses reconstruct. However, multiple examples of gene losses soon after duplication are also apparent. For instance, among flowering plants, *Amborella* alone may contain all the *GSK3* gene lineages descended from duplications along the ancestral branch to extant angiosperms. Gene expression data suggest that the Group I.1 genes have an evolutionarily conserved role in floral development, while members of other *GSK3* genes lineages have been independently recruited to reproductive roles, for example, a Group III gene in *Arabidopsis* and a Group IV gene in *Zamia*.

## Availability of supporting data

The data sets supporting the results of this article are available in the TreeBASE and Dryad repositories [http://purl.org/phylo/treebase/phylows/study/TB2:S14373 and [http://dx.doi.org/10.5061/dryad.76nr2, respectively].

## Methods

### Data retrieval, sequence alignments, and phylogenetic analysis

To reconstruct the phylogeny of the *GSK3* gene family, we searched five sequence databases for plant *GSK3* genes: the Phytozome (http://www.phytozome.net/), Ancestral Angiosperm Genome Project (AAGP; http://ancangio.uga.edu/), TIGR Plant Transcript Assemblies (http://plantta.jcvi.org/), 1KP project (http://www.onekp.com/), and NCBI nucleotide databases. NCBI’s dbESTs database was specifically searched for monilophytes, gymnosperms, asterids, and non-Poaceae monocots. The OneKP EST database was searched for *GSK3* genes from liverworts, mosses, lycophytes, and monilophytes to improve the sampling of these lineages. To identify *GSK3* homologs we used a reciprocal blast strategy: nucleotide sequences of *Arabidopsis GSK3* genes [10] were first used to seed tblastx searches to identify potential *GSK3* homologs in the above sequence data bases, and these were next used as queries in tblastx searches of all *Arabidopsis* genes. Only those genes with best hits to an *Arabidopsis GSK3* in the second blast search were considered to be true *GSK3* homologs. Some EST data were assembled into contigs, and ORFs were determined using Geneious Pro 5.4.6 [28] prior to phylogenetic analyses (see Additional file 1). ORFs covering less than 50% of complete genes were discarded. In total, we collected 445 *GSK3* genes from 67 species representing all major green plant lineages: green algae (2 species, 3 sequences), liverworts (2 species, 3 sequences), mosses (2 species, 8 sequences), lycophytes (2 species, 3 sequences), monilophytes (8 species, 51 sequences), gymnosperms (12 species, 73 sequences), and angiosperms (39 species, 329 sequences). Accession numbers for all sequences in their relevant databases are provided in Additional file 1.

Nucleotide sequences translation aligned using the MAFFT program [29] with the FFT-NS-i x1000 option in Geneious Pro 5.4.6 [28]. Maximum likelihood (ML) [30] phylogenetic analyses were conducted using RAxML 7.3.0 [31] with the GTRCAT model of evolution with bootstrap support calculated over 1000 replications. Sequences from the green algae *Chlamydomonas reinhardtii* and *Volvox carteri* were specified as outgroups. All phylogenetic analyses were performed on the University of Florida High Performance Computing cluster (http://hpc.ufl.edu/). Phylogenetic trees were viewed and edited with FigTree v1.3.1 (http://tree.bio.ed.ac.uk/software/figtree/). The AU test [17] for an alternative position of the single lycophyte clade was performed using CONSEL [32].

### Gene expression

Our data for *GSK3* gene expression are from global RNA-Seq analyses of transcriptomes assembled for *Amborella trichopoda*, *Aristolochia fimbriata*, *Liriodendron tulipifera*, *Persea americana*, *Nuphar advena*, and *Zamia vazquezii*, by the AAGP (Chanderbali et al. in prog.). For this study, data sets for each species were searched to obtain reads per kilobase per million mapped (RPKM) values [33] for each *GSK3* gene across multiple vegetative and reproductive tissues. For comparisons with *Arabidopsis GSK3* genes, normalized signal intensity values were obtained for corresponding tissues from the AtGenExpress microarray data set [27].

## Competing interests

The authors declare that they have no competing interests.

## Authors’ contributions

XQ conducted database searches, sequence alignments, and phylogenetic analyses. ASC performed the gene expression analyses. ASC, DES, PSS participated in the design of the study. All authors participated in the writing of the manuscript. All authors read and approved the final manuscript.

## Supplementary Material

Additional file 1 **Accession data and group membership of *GSK3 *homologs analyzed in this study.** Gene designations representing contigs constructed from multiple sequences have several accession numbers. Click here for file
